# Comparative Genomic Analysis of *Staphylococcus haemolyticus* Reveals Key to Hospital Adaptation and Pathogenicity

**DOI:** 10.3389/fmicb.2019.02096

**Published:** 2019-09-10

**Authors:** Maria Pain, Erik Hjerde, Claus Klingenberg, Jorunn Pauline Cavanagh

**Affiliations:** ^1^Pediatric Infections Research Group, Department of Clinical Medicine, UiT The Arctic University of Norway, Tromsø, Norway; ^2^Department of Chemistry, Norstruct, UiT The Arcic University of Norway, Tromsø, Norway; ^3^Department of Paediatrics, University Hospital of North Norway, Tromsø, Norway

**Keywords:** *Staphylococcus haemolyticus*, pangenome, multidrug resistance, bacterial genomics, pathogenicity, antibiotic resistance genes

## Abstract

*Staphylococcus haemolyticus* is a skin commensal gaining increased attention as an emerging pathogen of nosocomial infections. However, knowledge about the transition from a commensal to an invasive lifestyle remains sparse and there is a paucity of studies comparing pathogenicity traits between commensal and clinical isolates. In this study, we used a pan-genomic approach to identify factors important for infection and hospital adaptation by exploring the genomic variability of 123 clinical isolates and 46 commensal *S. haemolyticus* isolates. Phylogenetic reconstruction grouped the 169 isolates into six clades with a distinct distribution of clinical and commensal isolates in the different clades. Phenotypically, multi-drug antibiotic resistance was detected in 108/123 (88%) of the clinical isolates and 5/46 (11%) of the commensal isolates (*p* < 0.05). In the clinical isolates, we commonly identified a homolog of the serine-rich repeat glycoproteins *sraP*. Additionally, three novel capsular polysaccharide operons were detected, with a potential role in *S. haemolyticus* virulence. Clinical *S. haemolyticus* isolates showed specific signatures associated with successful hospital adaption. Biofilm forming *S. haemolyticus* isolates that are resistant to oxacillin (*mecA*) and aminoglycosides (*aacA-aphD)* are most likely invasive isolates whereas absence of these traits strongly indicates a commensal isolate. We conclude that our data show a clear segregation of isolates of commensal origin, and specific genetic signatures distinguishing the clinical isolates from the commensal isolates. The widespread use of antimicrobial agents has probably promoted the development of successful hospital adapted clones of *S. haemolyticus* clones through acquisition of mobile genetic elements or beneficial point mutations and rearrangements in surface associated genes.

## Introduction

*Staphylococcus haemolyticus* is an emerging pathogen of nosocomial infections, and the most frequently isolated coagulase-negative staphylococcal (CoNS) species alongside *Staphylococcus epidermidis* (Hope et al., 2008; Pereira et al., 2014; Nanoukon et al., 2017; Teeraputon et al., 2017). *S. haemolyticus* infections particularly affect immunocompromised patients and mainly occur as bloodstream and device-associated infections. Nosocomial *S. haemolyticus* isolates are ranked as the most antibiotic resistant species among the CoNS, and antibiotic therapy choices are therefore very limited (Hope et al., 2008; Kresken et al., 2011; Barros et al., 2012).

Compared to the more virulent *Staphylococcus aureus*, *S. haemolyticus* possesses few typical virulence factors (Takeuchi et al., 2005). Formation of biofilm (Fredheim et al., 2009; Giormezis et al., 2014; Pereira et al., 2014), production of phenol-soluble modulins (Da et al., 2017) and frequent phenotypic rearrangements due to a large number of insertion sequences (IS) (Takeuchi et al., 2005) have been suggested as important *S. haemolyticus* virulence determinants. However, these traits have not yet been linked explicitly to strains of clinical origin. The *oriC* environ is a chromosomal region of staphylococci proposed to be important for the evolution and differentiation of each staphylococcal species. There is little homology between the *oriC* environ of the different staphylococcal species, and the region does not contain genes essential for viability. The o*riC* environ is significantly larger in *S. haemolyticus* compared to that of *S. aureus* and *Staphylococcus epidermidis*. Moreover, almost half of the candidate coding sequences (CDS) for virulence are located within the *oriC* environ, encoding e.g., surface adhesins and capsular polysaccharides, factors that can modulate adherence and contribute to phagocytosis resistance (Takeuchi et al., 2005; Flahaut et al., 2008).

Despite the advancing clinical relevance of *S. haemolyticus*, knowledge about the transition from a commensal to an invasive lifestyle remains sparse. Moreover, there is a paucity of studies comparing pathogenicity traits between commensal and clinical invasive isolates. Predicting invasiveness of staphylococcal strains by use of marker genes is one approach to differentiate isolates with different pathogenicity. For *S. epidermidis*, the *ica* operon encoding biofilm formation and the insertion sequence element IS256; associated with aminoglycoside resistance, have been proposed as markers for invasive strains of *S. epidermidis* (Kozitskaya et al., 2004; Rohde et al., 2004). More recently, Méric et al. (2018) presented how calculation of a genotype risk score can predict pathogenicity in *S. epidermidis* isolates with 80% accuracy. With the advances in sequencing technologies and more available bacterial genomes new methods for analysis have emerged. The total number of genes in a bacterial population, collectively called the pan-genome, allows identification of genes more present and important in pathogenic strains (Tettelin et al., 2005). Studies of *S. haemolyticus* to date have focused predominantly on clinical isolates. The aim of this study was to identify factors important for infection and hospital adaptation by exploring the genomic variability of clinical and commensal *S. haemolyticus* using a pan-genomic approach.

## Materials and Methods

### Bacterial Isolates, Species Identification, Antibiotic Susceptibility and Biofilm Testing

This study includes 169 *S. haemolyticus* isolates; 123 clinical isolates and 46 commensal isolates. The clinical isolates (mainly from blood cultures, invasive catheters and wounds), in addition to five of the commensal isolates, were from different hospital units and had different geographical origins. They were collected between 1988 and 2010, and have been described previously (Cavanagh et al., 2014). The remaining 41 commensal isolates were skin samples isolated from healthy volunteers with no antibiotic exposure and no hospitalization or health care affiliation during the three previous months (Cavanagh et al., 2016). This was a separate collection from one geographical location (Tromsø, Norway) and collected between 2013 and 2014 (Cavanagh et al., 2016). An overview of all the isolates used and their characteristics can be found in Supplementary Data S1. Species identification was done by 16s rRNA sequencing and/or matrix-assisted laser desorption ionization time-of-flight mass spectrometry (MALDI-TOF MS) using a Microflex LT instrument (Bruker Daltonics, Bremen, Germany), Flex Control software and the MALDI Biotyper 3.1 software (Bruker Daltonics, Bremen, Germany). Antibiotic susceptibility testing was performed as previously described (Cavanagh et al., 2014, 2016), and interpreted according to the 9th version of the EUCAST guidelines^1^. Isolates resistant to three or more classes of antibiotics were classified as multidrug resistant (MDR). Semi-quantitative determination of biofilm formation was performed with the modified Christensen assay, as described previously (Fredheim et al., 2009). Isolates were considered biofilm positive if they had an optical density (OD) value >0.2 above the negative control.

### Whole Genome Sequencing (WGS), Assembly and Annotation

The WGS procedure for all clinical isolates and five of the 46 commensal isolates is described in a previous study (Cavanagh et al., 2014). For the remaining 41 commensal isolates, bacterial DNA was extracted and prepared for WGS using the Wizard Genomic DNA purification kit (Promega, Madison, United States) according to the manufacturer’s instructions. WGS was performed on index-tagged libraries for each *S. haemolyticus* strain by paired-end sequencing at the Norwegian Sequencing Centre on an Illumina MiSeq (Illumina Inc., San Diego, California, United States). Subsequently, all 169 genomes were (re-assembled using SPAdes version 3.7 software (Bankevich et al., 2012), with some modification to the default parameters. Contigs >500 bp were reordered relative to the only complete fully annotated closed reference genome (JCSC 1435) (Takeuchi et al., 2005) using ABACAS (version 1.3.1). Protein CDSs were predicted using Prokka v1.12 (Seemann, 2014) using default settings. The sequences are deposited in the European Nucleotide Archive^2^; study accession no ERP000943 and ERP114853.

### Phylogeny and Molecular Typing

Subtyping of the 169 isolates was performed using kSNP3 to identify single-nucleotide polymorphisms (SNPs) in the core genomes and to reconstruct a parsimony phylogenomic tree (Gardner et al., 2015). Phylogenetic trees were visualized using the online tool iTol^3^.

### Pan-Genome and Pan-Genome-Wide Association Study (Pan-GWAS) of *S. haemolyticus*

Pan-genome analyses of all 169 isolates were performed using the Roary software package with default settings (Page et al., 2015). The program generates a file containing all the predicted gene clusters and the sequence identifier of each isolate containing said gene. Based on this file, core, accessory and unique genes were extracted and saved as individual lists. The accessory list was subdivided based on clusters common for both clinical and commensal isolates, in addition to clusters unique to each group. The unique list was further subdivided based on genes identified in clinical and commensal isolates. These lists were uploaded to eggNOG to get cluster of orthologs groups (COG) identifications (Huerta-Cepas et al., 2016).

Files created by Roary, were used as input for Scoary, a microbial pan-GWAS tool that calculates the association between all genes in the accessory genome and traits defined by the users. For our purpose we only used one trait; whether a given isolate were of clinical or commensal origin. Based on this information Scoary reported a list of genes sorted by strength of association per gene (Brynildsrud et al., 2016).

### *In silico* Analysis and Statistics

The resistance gene identifier in the comprehensive antibiotic resistance database (CARD; version 1.1.1; Department of Biochemistry and Biomedical Science; McMaster University, Canada (Jia et al., 2017) was used to predict genes presumed to confer antibiotic resistance, and the results were further compared with the phenotypic susceptibility test results. Potential virulence factors were identified by homology searches against the virulence factor database (VFDB) together with putative virulence factors previously predicted by Takeuchi et al. (2005) and Chen et al. (2016). Identification of IS elements was performed using the ISsaga program (Varani et al., 2011). The presence of IS elements were confirmed by sequence search against the complete *S. haemolyticus* sequence collection (both on contigs and CDS). Sequence coverage of contigs harboring IS elements was used to quantify the copy numbers: the coverage of contigs with IS elements divided by the overall average coverage of its respective genome. Identification of putative plasmids was performed by screening the genome assemblies for plasmid replicon (*rep*) genes using the PlasmidFinder database (Carattoli et al., 2014) with coverage settings set to default of 75%. Identification of prophages, potentially important for horizontal gene transfer (HGT), was performed by using PHASTER (Arndt et al., 2016). Plasmid replicon sequences with more than 80% coverage and predicted intact phages identified in more than 10 isolates were further investigated by sequence search in order to confirm the presence of plasmid and phage genes, respectively.

Data were also analyzed using IBM-SPSS statistical software (IBM Corp. Released 2015. IBM SPSS Statistics for Windows, Version 23.0. Armonk, NY: IBM Corp.). Categorical data are displayed as ratios and frequency (%), and analyzed using the Chi square test. Pathogenicity is a complex multifactorial property. We used biological knowledge to identify known pathogenicity-associated traits enriched in clinical isolates. In particular, we focused on antibiotic resistance genes (ARGs) and previously established virulence factors. We developed different scores including the following four traits; *aacA-aphD*, *mecA*, *folP* and phenotypic biofilm production. In order to find a pragmatic score that could differentiate between a clinical and a commensal isolate we calculated the area under receiver operating characteristic (ROC) curves, and its 95% confidence interval.

## Results

### Genome Composition and Genetic Variability

The average size of the assembled genomes was 2.52 Mb (2.32–2.86 Mb), with an average of 120 (32–415) contigs per genome. Each genome had on average 2,457 (2,239–2,816) predicted protein sequences (CDSs) with an average GC content of 32%.

**FIGURE 1:**
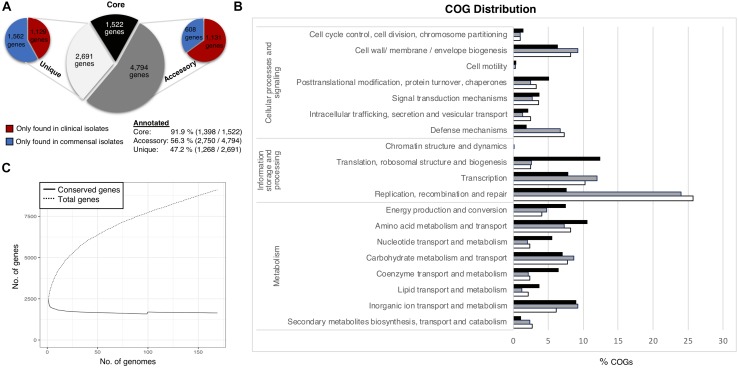
*Staphylococcus haemolyticus* pangenome statistics. **(A)** The size and distribution of the pangenome into the subgroups; core (shared by all isolates), accessory (shared by some isolates) and unique (found only in one isolate). **(B)** Collective distribution of core (black), accessory (gray) and unique (white) genes in COG. **(C)** Pangenome curve generated by plotting total number of gene families in the pan and core genome.

The pan-genome of the 169 *S. haemolyticus* isolate dataset comprised 9,092 Cluster of Orthologous groups (COGs). We divided the pan-genome into core genes (genes shared by all strains), accessory genes (genes shared by some but not all strains) and unique genes only present in one genome (Figure 1A). The pan-genome sub-groups were annotated and sorted into COG categories (Figure 1B). Gene accumulation curves showed that the core genome plateaued at 1,522 genes reflecting a stable core and an open pan-genome where the addition of each new genome increases the total gene pool (Figure 1C).

Thirty-two percent of the annotated genes were categorized as function unknown, and this class was excluded from the graphical representation of the COG categories. The most abundant categories in the core genome were genes involved in housekeeping functions, like transcription and translation, and different metabolism categories. The accessory genome had a larger portion of genes associated with mobile genetic elements (MGE) such as transposons and bacteriophages (transcription, replication, recombination and repair (24%), which was also the most enriched category amongst the unique genes (25.7%). Genes involved in transcription (12%), genes associated with cell wall and membrane biogenesis (9.2%) and defense mechanisms (6.8%) were also considerably more prevalent in the accessory and unique gene pool, compared to the core gene pool. No significant differences of the COG distribution were found between the clinical and commensal group (data not shown).

### Phylogeny and Population Structure

**FIGURE 2:**
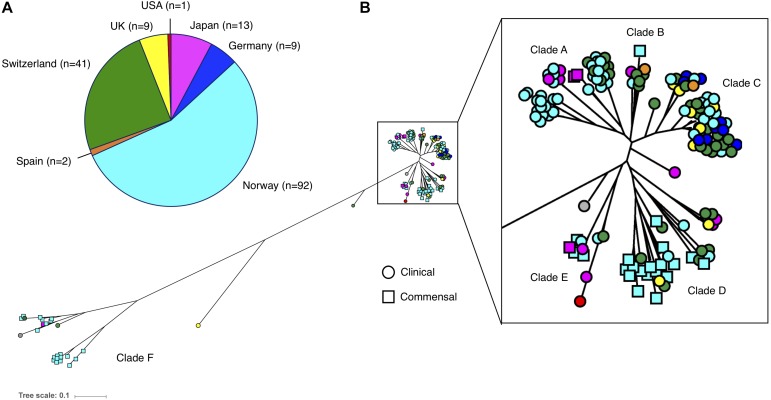
**(A)** Number and origin of isolates included in this study. **(B)** Phylogenetic tree of the 169 *S. haemolyticus* isolates, based on SNPs in the core genome. Each isolate is color coded based on country of origin, as demonstrated in the pie chart. Clinical isolates are displayed as circles and commensal isolates as squares.

The phylogenetic reconstruction grouped the 169 isolates into six clades (A–F). There was a distinct distribution of clinical and commensal isolates into the different clades (Figure 2). The two largest clades, A and C, consisted almost exclusively of clinical isolates (88/90; 98%), while the majority of the commensal isolates (39/46; 85%) were found in clades D and F. The clinical isolates in clade A–C were more closely related compared to the commensal isolates. Twenty-eight of 123 (22.8%) clinical isolates were grouped in the three clades (D–F) predominantly consisting of commensal isolates. A long branch separating the “commensal heavy” clade F (78% commensal isolates) from the rest of the isolates underlines the high variability observed in the commensal isolates, compared to the clustered clinical isolates (Figure 2). We observed that with the exception of clade F, the isolates in all the other clades were closely related, independent of country of origin. The commensal isolates in clade F, mainly originating from the same geographic location in Norway, were in contrast very diverse.

### Antibiotic Resistance and Mobile Genetic Elements (MGE)

**FIGURE 3:**
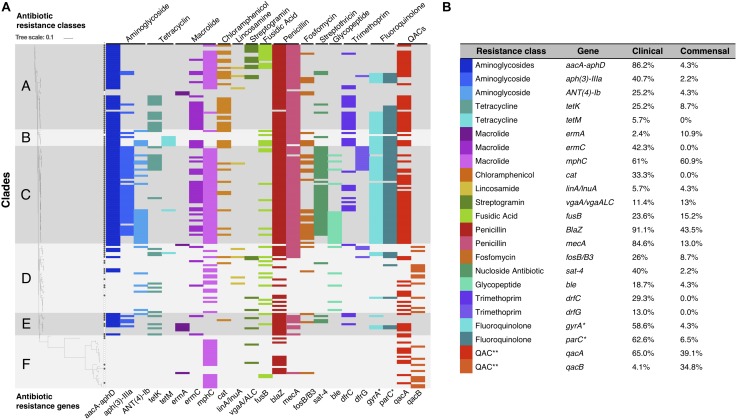
**(A)** Graphical representation of *S. haemolyticus* antibiotic resistance genotype plotted onto the phylogenetic SNP-based core tree. The figure shows the presence and absence of ARGs and classes across the different clades. ^∗^Mutations in *gyrA* and *parC* gives resistance to fluoroquinolones. ^∗∗^ QACs: quaternary ammonium compounds. **(B)** The percentage of each ARG present in the clinical and commensal subgroups.

Phenotypically, 113/169 (68%) of the isolates were classified as MDR; 108/123 (88%) of the clinical isolates (Cavanagh et al., 2014) and 5/46 (11%) of the commensal isolates (Cavanagh et al., 2016) (*p* < 0.05). Using a genotypic approach, we identified the ARGs for most of the observed phenotypes, and overall, phenotypic and genotypic resistance correlated well. However, ARGs toward eight additional antibiotic classes were also detected, but not phenotypically tested (Figure 3).

ARGs were among the most common genes among all clinical isolates. In particular *mecA*, *aacA/aphD, blaZ* were predominantly found in invasive isolates (Table 1). Phenotypic macrolide resistance was common in both the commensal (30/46; 65%) and the clinical (100/123; 81%) isolates. However, the majority of commensal isolates carried the macrolide resistance gene *mphC* (28/46; 61%) and few carried *ermA* (5/46; 11%), while the clinical isolates had both *mphC* (75/123; 61%) and *ermC* (52/123; 42.3%). The antiseptic resistance genes *qacA* and *qacB* were found in both clinical and commensal isolates, however *qacB* was identified predominantly in the commensal isolates whereas *qacA* was detected predominantly in clinical isolates (Figure 3).

**TABLE 1:** Genes and gene versions enriched in 123 clinical isolates, sorted by significance.

**Gene name**	**SH_ref**	**Annotation**	**Clinical (%)**	**Commensal (%)**
NA	SH1612	Acetyltransferase GNAT	86.2	4.4
aacA-aphD	SH1611	Bifunctional AAC/APH	85.4	4.4
folP^X^	SH2496	Dihydropteroate synthase	78.9	0.0
folB^X^	SH2495	Dihydroneopterin aldolase	78.1	0.0
NA	SH2607	AtP-binding protein (plasmid)	76.4	2.2
paaZ	SH0090	Bifunctional protein PaaZ	85.4	13.0
mecA	SH0091	Methicillin resistance gene	85.4	13.0
ugpQ	SH0089	Glycerophosphodiester phosphodiesterase	82.1	13.0
blaR1^X^	SH1763	Regulatory protein BlaR1	84.6	19.6
tagE	SH2252	Truncated glycosyltransferase	86.2	21.7
blaZ^X^	SH1764	Beta-lactamase	78.1	15.2
blaI^X^	SH1762	Penicillinase repressor	60.2	2.2
ansA^X^	SH1433	Putative L-asparaginase	55.3	0.0
NA^X^	SH2156	Ferrichrome ABC transporter truncated	88.6	30.4
nikA^X^	SH0292	Nickel-binding periplasmic protein	94.3	41.3
fabG^X^	SH0438	FabG	89.4	32.6
NA	SH0155	MFS transporter	96.8	47.8
sraP^X^	SH0326	SraP	96.8	47.8
secA2^X^	SH0331	Protein translocase subunit SecA2	96.8	47.8
yjjG^X^	SH0452	Pyrimidine 5’-nucleotidase YjjG	84.6	26.1
PHAGE		StauST398-2/vB_saus_phi2	51.2	0.0

*All these genes/gene versions were significantly enriched in clinical isolates (*p* < 0.001), the order of presentation from top of the table indicates those with the lowest *p* values. ^*X*^ These genes exist in different conserved versions in the clinical and commensal isolates. SH_ref; *S. haemolyticus* reference gene numbers from Takeuchi et al. (2005), accession number AP006716.1. The complete list of genes can be found in Supplementary Data S2.*

For the plasmid analysis, we found a total of 51 replicon sequences, with an average of 5.5 replicon per clinical isolates and 7.6 average replicon in commensal isolates. When a replication sequence was identified in more than 10 isolates, we examined the presence of the entire plasmid. For smaller plasmids presence was easily determined, and numbers in clinical and commensal isolates were calculated and presented in Table 2. Larger plasmid sequences were often split over several contigs, thus not all of the plasmid genes were identified. If the whole plasmid could not be identified, we looked for the presence of its cargo genes instead. The replicon sequence of *S. haemolyticus* plasmid pSHaeB, carrying *ermC*, was only found in the clinical isolates (55/123; 45%). The *S. aureus* plasmid replicon for pUB110, carrying kanamycin and bleomycin resistance genes, was identified in 23/123 (19%) clinical and 2/46 (4%) commensal isolates. Chloramphenicol resistance plasmid pC221 was predicted based on the replicon sequence, and the chloramphenicol gene (*cat*) was identified in 39 isolates, all of clinical origin. In addition, the plasmids pSHaeA and pSHAaeC, the former carrying the fosfomycin resistance gene, were identified in our collection but in less than 1/3 of all isolates, and with no clear difference between clinical and commensal isolates. Several larger plasmids were predicted based on replicon sequence but none of these were identified in their entirety. We extracted what we considered interesting cargo genes from these plasmids and determined the prevalence of these (Table 3).

**TABLE 2:** Smaller plasmids and their distribution in clinical (*n* = 123) and commensal (*n* = 46) isolates.

**Plasmid**	**Origin**	**Size**	**Associated genes**	**Clinical (%)**	**Commensal (%)**
pSHaeA	*S. haemolyticus*	2,300 bp	*fosB* (fosfomycin resistance)	26.8	8.7
pSHaeB	*S. haemolyticus*	2,366 bp	*ermC* (erythromycin resistance)	44.7	0
pSHaeC	*S. haemolyticus*	8,180 bp	MFS transporter, *mobC*, *marR* transcriptional regulator	8.9	6.5
pWBG1773	*S. aureus*	2,916 bp		25.2	28.3
pKH21	*S. aureus*	2,531 bp	Lincosamin resistance	5.7	4.3
pWBG754	*S. aureus*	2,241 bp	*qacC*	29.3	17.4
pC221	*S. aureus*	4,555 bp	Chloramphenicol resistance	31.7	0
pUB110	*S. aureus*	4,548 bp	Bleomycin and kanamycin resistance	18.7	4.3

**TABLE 3:** Distribution of genes associated with larger plasmids in the isolates.

**Associated plasmids**	**Origin**	**Gene**	**Clinical (%)**	**Commensal (%)**
pSE-12228-05, pWBG753	*S. epidermidis S. aureus*	Tn552	72.4	6.5
pSE-12228-05, pWBG753	*S. epidermidis S. aureus*	blaR1	91.9	43.5
pSE-12228-05	*S. epidermidis*	Penicillinase repressor	94.3	45.7
pWBG753	*S. aureus*	blaZ	91.9	43.5
VRSAp	*S. aureus*	aacA-aphD	85.3	4.3
VRSAp	*S. aureus*	qacA/B	69.1	73.9
VRSAp	*S. aureus*	qacR	67.5	76.1
pWBG753	*S. aureus*	tetK	26	8.7
SAP099B, pLEW6932	*S. aureus*, *Staphylococcus* sp.	cadC	17.1	17.4
SAP099B, pLEW6932	*S. aureus*, *Staphylococcus* sp.	arsR	4.1	63

We predicted 13 different intact prophages in our collection, identified in 68% (114/169) of the isolates (clinical 94/123; 76% and commensal 17/46; 37%). Five of these prophages were found in more than 10 isolates. The most prevalent phage, exclusively found in only clinical isolates (63/123; 51%), was predicted as staphylococcal phage vB_Saus_phi2 and *S. aureus* phage StauST398-2. Sequence searches of the two phage genes matched the same *S. haemolyticus* genes. Staphylococcus phage SPbeta-like is a phage of 127,726 bp with 156 CDS, and was identified in 21 clinical isolates and in one commensal isolate. This phage carries the genes *aacA-aphA* and *dfrC*, encoding resistance to gentamicin and trimethoprim, in addition to five IS256 and one IS431 element (CDS 146–156). Of all the isolates carrying this phage, we could identify the first 145 genes in the same order as in the reference phage.

We identified a large number of IS elements, ranging from 15 to 88 per each isolate. ISSha1 and IS1272 were found as several copies in all isolates. IS256 was found almost exclusively in clinical isolates (106/123; 86%) and rarely (5/46; 11%) in commensal isolates. The transposon Tn552/IS481 was also identified predominantly in clinical isolates (89/123; 72%) compared to commensal isolates (6/46; 13%).

### Biofilm Production and Biofilm Encoding Genes

Phenotypic biofilm production was significantly more prevalent among clinical isolates (83/123; 67%) versus commensal isolates (16/46; 35%), *p* < 0.001. However, we did not detect *ica* genes which typically encode biofilm production in other CoNS species.

### Putative Surface Proteins

The C-terminal part of a hypothetical serine-rich surface protein (SH0326) named *sraP* (serine rich adhesin for platelets) was present in 119/123 (97%) of the clinical isolates and in 22/46 (48%) of commensal isolates. The partial *sraP* gene was located up-streams of *secY2*, the first gene in the accessory sec system (aSec); a structure dedicated to transport and modification of this surface protein. The N-terminal was located on a different contig which was, in most cases, correctly assembled directly upstream of the *aSec* contig. The central part of *SraP*, known to contain long stretches of repeated patterns was lost during assembly. Mapping sequence reads on assemblies of isolates, predicted to contain *sraP*, against the full length SH0326 showed that these strains did indeed have the full sequence. However, there appeared to be individual differences in the length of these repeat sequences. The complete *aSec* operon was identified in 119 clinical isolates (97%) and 34 (74%) of commensal isolates by performing sequence read mapping. Isolates without *sraP* and *aSec* belonged to the commensal clades D (12 isolates) and F (3 isolates), and one commensal isolate in clade B.

### Putative Capsule Polysaccharide (CP) Operons

**FIGURE 4:**
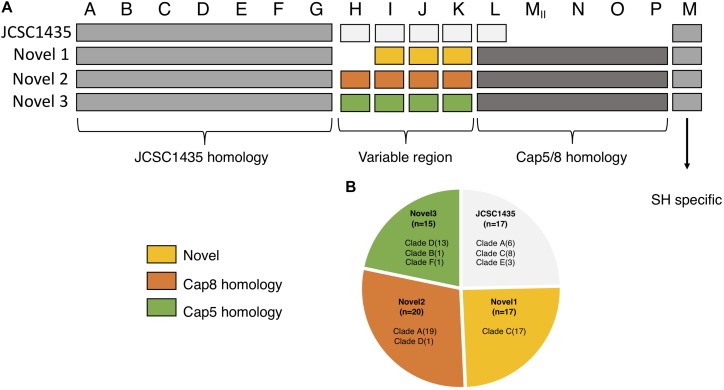
Organization of the different polysaccharide capsules (CP) operons identified in our collection. **(A)** The three novel varies in their homology to the CP in JCSC1435. capA-G is homologs among all isolates with identified capsule operon. The region capH-K is what separates the four types from one another; novel 1 with no known homology, novel 2 shows homology to *S. aureus cap8* and novel 3 with homology to *S. aureus cap5*. The region capL-P is homologs to *S. aureus* and is found in all three novel types, a region absent in JSCS1435. **(B)** Pie chart showing the distribution of all identified CP operons in this study.

We identified four different structures of the CP operons among the 169 isolates. Seventeen isolates, all of clinical origin, had the *capA-M* operon structure similar to that described in *S. haemolyticus* JCSC 1435 (Takeuchi et al., 2005). Another 52 isolates had three potential novel CP operons (Figure 4). These three novel CP operons were homologs to the *capA-G* of *S. haemolyticus* JCSC 1435 (65–100% CDS identity). Each novel CP operon contained a *capH-K* region unique to its group, followed by the region *capM_II_-P* (CDS 51–78% identity) which was common to all the three novel versions. The last gene in the operon, named *capM* in JCSC1435 was present in all four CP operon versions. *capM* identified in the three novel versions were named *capM_II_* to distinguish it from the already annotated *capM* found in JCSC 1435. *capL-M_II_* shared homology with *S. aureus* cap5/8. The novel CP version 1 was lacking the *capH* gene and *capI-K* did not show considerable homology to any other known CP genes. However, the novel CP version 2 *capH-K* showed homology to *S. aureus* cap8 (CDS 55–72% identity) and novel CP version 3 to *S. aureus* cap5 (CDS 57–68% identity).

### Genes and MGE Significantly Different Between Clinical and Commensal Isolates

In order to identify a specific signature in the gene repertoire of the clinical isolates important for hospital adaptation, a pan-GWAS analysis was performed on the accessory genome. 1887 predicted genes were statistically different between the two groups (*p* < 0.05), and the genes found most enriched for each group are listed in Tables 1, 2. As the population of *S. haemolyticus* is diverse, the gene sequence of genes with the same function varied significantly between the isolates, resulting in several orthologs clusters for one gene. Two enzymes in the folate pathway, dihydropteroate synthase (*folP*) and dihydroneopterin aldolase (*folB*) are a representative example of this. One version was only present in clinical isolates (96/123 isolates, 78%), and not found in any commensal isolates. A different version was found in all 46 commensal isolates, and 27/123 (22%) of clinical isolates. The majority of the clinical isolates of the latter group belonged to the commensal clade D and F (19 isolates), with 8 isolates scattered amongst clade A, B and E. Extracting these CDSs from each isolate and aligning them displays conserved differences between the genes in the two groups.

**FIGURE 5:**
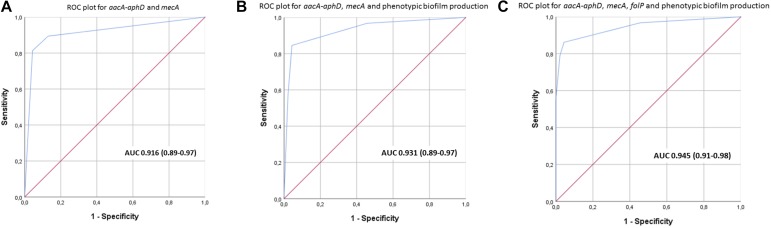
**(A–C)** Receiver operating characteristics (ROC) curves with area under curve (95% confidence interval) for scores using different combinations of *aacA-aphD*, *mecA*, *folP* and phenotypic biofilm production in order to discriminate between clinical and commensal isolates.

The most prevalent genes with a known function enriched in the clinical isolates included several ARGs (Table 1). The most prevalent genes with a known function enriched in the commensal isolates included several genes involved in metal control, transposases, and generally different versions of genes found in clinical isolates (Table 4). Using scores including one or more ARGs and phenotypic biofilm formation resulted in ROC curves with area under the curve (AUC) values >0.9, indicating a high discriminatory capacity to differentiate between clinical and commensal isolates (Figure 5).

**TABLE 4:** Genes and gene versions enriched in 46 commensal isolates, sorted by significance.

**Gene name**	**SH_ref**	**Annotation**	**Clinical (%)**	**Commensal (%)**
folB^X^	SH2495	Dihydroneopterin aldolase	22.0	100.0
folP^X^	SH2496	Dihydropteroate synthase	23.6	100.0
group_2286		Major Facilitator (MFS)	0.0	56.5
csoR		Copper-sensing transcriptional repressor CsoR	8.1	76.1
copZ		Copper chaperone CopZ	8.1	73.9
copA		Copper-exporting P-type ATPase	8.1	71.7
czcD		Cadmium, cobalt and zinc/H(+)-K(+) antiporter	8.1	71.7
NA^X^	SH0287	Caax amino terminal protease family protein	4.1	63.0
arsR		ArsR family transcpriptional regulator	4.1	63.0
ISSha1^X^	SH2073	Transposase	0.0	47.8
grxC		Glutaredoxin	8.1	69.6
nikA^X^	SH0292	Nickel-binding periplasmic protein	4.1	60.9
ydaF^X^	SH2658	Putative ribosomal *N*-acetyltransferase YdaF	7.3	65.2
recQ	SH1430	ATP-dependent DNA helicase RecQ	44.7	100.0
ansA^X^	SH1433	Putative L-asparaginase	45.5	100.0
IS30		IS30 family transposase	1.6	47.8
yjaB		Putative N-acetyltransferase YjaB	2.4	50.0
group_1785		Putative Insertion element/transposase	2.4	50.0
IS1272^X^	SH2041	IS1272	0.0	39.1
yodJ ^X^	SH0186	Putative carboxypeptidase YodJ	2.4	47.8

*All these genes/gene versions were significantly enriched in commensal isolates (*p* < 0.001), the order of presentation from top of the table indicates those with the lowest *p* values. ^*X*^ These genes exist in different conserved versions in the clinical and commensal isolates. SH_ref; *S. haemolyticus* reference gene numbers from Takeuchi et al. (2005), accession number AP006716.1. The complete list of genes can be found in Supplementary Data S2.*

## Discussion

There is a significant lack of knowledge about *S. haemolyticus* pathogenicity. In the present comparative genome study of clinical and commensal isolates we identified several genetic determinants and genotypes associated with the pathogenicity and the success of *S. haemolyticus* in the hospital environment. Resistance to commonly used antimicrobial agents and disinfectants, in addition to genes encoding proteins involved in adhesion and human immune defense escape predominates in the clinical isolates.

### Population Structure – Distinct Signatures of Clinical and Commensal Isolates

The establishment of hospital adapted *S. haemolyticus* clones was previously reported by our group (Cavanagh et al., 2014). In the current study the majority of the clinical and commensal isolates form distinct phylogenetic groups when phylogeny is reconstructed based on SNPs in the core genome. In clades where clinical isolates are grouped with commensal isolates, they display a signature more similar to the commensals. In *S. epidermidis* there has been more research on phylogenetic relationship between clinical isolates, but data reported are conflicting. Conlan et al. (2012) showed that commensal and invasive isolates were grouped as distinct groups. In contrast, Méric et al. (2018) recently demonstrated that pathogenic clones of *S. epidermidis* can arise from various commensal backgrounds. This was shown by the absence of specific disease associated clades in a comparative study of invasive and commensal isolates. *Staphylococcus haemolyticus* and *S. epidermidis* are the two most recovered CoNS species from infection. Abundance of *S. epidermidis* on skin enhances the probability of contamination of indwelling devices. While *S. haemolyticus* also is a skin commensal, the population structure observed among our isolates suggests that specific persistent hospital adapted clones of *S. haemolyticus* are the major sources of infections, and that skin contamination is less likely (Tognetti et al., 2012; Kaspar et al., 2016; Byrd et al., 2018).

Surveillance and molecular typing are important in order to monitor the epidemiology of established and developing *S. haemolyticus* hospital clones. The availability of online resources, such as PubMLST^4^, Enterobase^5^, and BacWGSTdb^6^, for bacterial typing offers rapid classification and source tracing, which is increasingly important in a globalized community (Ruan and Feng, 2016; Alikhan et al., 2018; Jolley et al., 2018; Ruan et al., 2019). Currently, it is possible to investigate *S. haemolyticus* epidemiology using the traditional MLST database^7^, but extended MLST schemes are unavailable. As *S. haemolyticus* is an emerging nosocomial pathogen with extended antibiotic resistance, an online resource offering rapid typing and phylogenetic relatedness linked to antibiotic resistance genes and clinical data would be very useful.

The accessory gene repertoire of the clinical isolates is characterized by high prevalence of ARGs. This was expected as *S. haemolyticus* infections are commonly caused by MDR isolates (Barros et al., 2012; Chang et al., 2018). Creating a score with ARGs and phenotypic biofilm formation clearly separated the invasive from the commensal isolates. This can be useful in clinical microbiology when conveying results of a positive *S. haemolyticus* culture back to the treating clinician. Biofilm forming *S. haemolyticus* isolates that are resistant to oxacillin (*mecA*) and aminoglycosides (*aacA-aphD)* are most likely invasive isolates whereas absence of these traits strongly indicates a commensal isolate.

The interpretation of the *folB/folP* results is complex. We found distinct conserved differences in *folB* and *folP* clearly separating clinical from commensal *S. haemolyticus* isolates. In *S. aureus* five mutations in *folP* have been shown to directly contribute to sulfonamide resistance (Griffith et al., 2018). Combination treatment of trimetoprim-sulfamethoxazole has been used in infections caused by methicillin resistant staphylococci, and the rates of trimetoprim-sulfamethoxazole resistance is increasing (Khamash et al., 2018). Neither of the two versions of *folP* that we identified in our *S. haemolyticus* collection had the mutations shown to increase resistance to sulfonamides in *S. aureus*. However, there is substantial sequence variations between the *S. haemolyticus* and *S. aureus folB* and *folP* genes, and other mutations could also lead to decreased susceptibility. The importance of the *folP* versions in *S. haemolyticus* needs further evaluation, and extensive testing on sulfonamide resistance is needed.

In the successful epidemic *S. aureus* EMRSA-15, *mecA* acquisition, mutations in *gyrA* and *glrA;* resulting in fluoroquinolone resistance, and uptake of plasmids carrying *ermC*, was shown to be strong drivers of evolution (Holden et al., 2013). Uptake of plasmid pSHaeB carrying *ermC* was only observed for the clinical *S. haemolyticus* isolates, and has also been shown to be prevalent in several clinically important staphylococcal species (Águila-Arcos et al., 2017). The observed population structure in *S. haemolyticus* appears more similar to *S. aureus* than to *S. epidermidis.* In *S. aureus* defined pathogenic clones have adapted to the specific challenges of the hospital environment (Rasmussen et al., 2013), and our data support that similar phenomenon occurs in *S. haemolyticus*.

IS256, found in the majority of clinical *S. haemolyticus* isolates, has also been reported to be more common in nosocomial isolates of *S. epidermidis*, and has been associated with gentamicin resistance due to co-localization on the transposon Tn4001, in addition to biofilm formation (Kozitskaya et al., 2004; Conlan et al., 2012; Sharma et al., 2018). IS256 can shape the genome by affecting gene expression. In several successful virulent MRSA STs, such as ST 247, the presence of IS256 has been linked to increased virulence, vancomycin resistance and formation of small-colony variants (Kleinert et al., 2017). The opposite was demonstrated in Brazilian MRSA ST 239 isolates where IS256 was integrated near global regulatory genes (*agr* and *mgr*) likely leading to rapid changes of bacterial traits, resulting in reduced virulence (Botelho et al., 2019). IS256 has been suggested as a marker for molecular typing and identification of nosocomial, invasive *S. epidermidis* isolates (Gu et al., 2005; Yao et al., 2005; Montanaro et al., 2007). There is also some evidence indicating that IS256 is not only enriched within invasive isolates, but also more prevalent in isolates with poor treatment outcome (Post et al., 2017).

The transposon Tn552/IS481, was also predominantly identified in clinical *S. haemolyticus* isolates, and this MGE often carry antiseptic resistance and beta lactamase genes (Anthonisen et al., 2002). The pandemic *S. aureus* ST239 has acquired resistance to multiple antibiotics and antiseptics, among them *qac A* and *B* (Chang et al., 2017). Qac proteins are efflux pumps that protect bacteria not only from a variety of toxic substances but also from fluorquinolones and beta-lactams (Prag et al., 2014; Wassenaar et al., 2015; Taheri et al., 2016). In our study, *qacA* was detected more often in clinical isolates, while *qacB* was almost exclusive to the commensal isolates. *qacA* has been reported to have a broader spectrum of resistance than *qacB*, and this might be the reason why we found *qacA* more often in the clinical samples (in 65% clinical isolates and 39% commensal isolates), obtained from an environment with higher antibiotic pressure (Wassenaar et al., 2015).

### The Adhesion Protein SraP Is Highly Prevalent in Clinical Isolate

In the clinical isolates we commonly identified a homolog (SH0326) of the serine-rich repeat glycoproteins SraP of *S. aureus* and the accessory sec system, dedicated for the export of SraP. SraP belongs to a highly conserved family of serine-rich surface glycoproteins of Gram-positive bacteria. Expression of SraP has been linked to adhesion to different types of cells, including human platelets and is associated with infective endocarditis (Siboo et al., 2005; Yang et al., 2014; Bensing et al., 2016). The higher prevalence of the *aSec* system and *sraP* is likely advantageous for the clinical *S. haemolyticus* isolates as they are mainly isolated from blood. In *S. epidermidis*, genome signatures linked to pathogenicity identified the aSec gene *asp3* as one of four strong virulence predictors. In their full list of pathogenicity-associated signature genes *sraP* was also identified (Méric et al., 2018).

### A Novel Capsular Polysaccharide Operon

The polysaccharide capsule (CP) in *S. haemolyticus* has been shown to play a role in the protection against uptake and killing by human neutrophils (Flahaut et al., 2008). In *S. aureus* it was demonstrated to modulate adherence to endothelial surfaces *in vitro*, and to promote bacterial colonization and persistence on mucosal surfaces in animal models (Riordan and Lee, 2004).

In this study we found four different capsule operons of which only the first type has been described in *S. haemolyticus* JCSC1435 previously (Takeuchi et al., 2005). Two of the three novel CP (*capNOP*) structures have homology to the *S. aureus* cap 5/8 genes, and identification of *capO* in *S. haemolyticus* has not previously been reported. The third type has no homology to any previously described cap version. The new CP versions in *S. haemolyticus* have a variable region, capH-K which is unique to each of the four versions with little or no homology between them. The GC content of this variable region is also significantly lower than the surrounding cap genes, indicating the variable region was acquired by HGT. Both CP genes and SCC are located in the *oriC* environ, a region of high genomic flexibility, allowing staphylococci to maintain or acquire genes needed for the adaption to on-going environmental changes (Hiramatsu et al., 2014). The new CP structures were mainly found in isolates belonging to two clinical clades. The four different CP types were shown to be clade specific, which has also been shown for *S. aureus*. In *S. aureus* 13 putative capsular operons have been reported, but only CP type 5 and 8 have been associated with disease (Mohamed et al., 2019), thus the three novel CP types now identified in *S. haemolyticus* need to be further investigated for their role in virulence.

Taken together, our findings point toward HGT as a driving force in *S. haemolyticus* evolution and in response to the selective pressure of broad-spectrum antibiotics used in hospitals.

### The *S. haemolyticus* Pan-Genome Distribution Reflects the High Variation of Commensal Isolates

The pan-genome analysis reflected a relatively stable core genome which is comparable to what is observed in *S. epidermidis*, *S. aureus* and *Staphylococcus lugdunensis*. In *S. haemolyticus* the core genome is slightly smaller which could be explained by the higher number of unique genes in the commensal isolates (Méric et al., 2015, 2018; Argemi et al., 2018). Similar to *S. haemolyticus*, *S. aureus* and *S. epidermidis* also have an open pan-genome in contrast to *S. lugdunensis* (Bosi et al., 2016; Argemi et al., 2018). However, the pan-genome accumulation curves for *S. epidermidis* and *S. aureus* are not as steep as we see for *S. haemolyticus* (Conlan et al., 2012; Méric et al., 2015; Sharma et al., 2018). The relatively large pan-genome of *S. haemolyticus* is, at least in part, due to the variation seen in genes of the same function, resulting in two or more clusters for one gene. The most abundant categories in the core genome were genes involved in housekeeping functions, like transcription and translation, and different metabolism categories, a result similar to reports on *S. aureus* and *S. epidermidis* (Bosi et al., 2016; Sharma et al., 2018). The large repertoire of genes in the accessory genome confer advantages in highly variable environmental conditions.

### Strengths and Limitation With the Study

This study is the largest comparative study of clinical and commensal *S. haemolyticus* isolates to date, and the clinical isolates have wide spatial and temporal distribution. We have used state of the art technology to analyze pathogenicity traits and the genetic signatures of clinical and invasive isolates. The study also has limitations. First, the majority of the commensal isolates are from one geographic location, and more recently collected than the invasive isolates. Still, the very diverse commensal population indicates that commensal isolates may lack the pathogenic traits of hospital-adapted clones. Second, the invasive isolates are collected over a wide time-span and some of the isolates originated from infections two decades ago. Still we see a very clear phylogenetic clustering, and invasive isolates from different geographical origin cluster together. We believe this clearly indicates the emergence of disease-causing isolates with a homogenous genetic signature. Third, it would have been of interest to collect skin samples from hospitalized patients without infection, in order to see whether colonization of more pathogenic isolates emerges after hospitalization. We did not have the opportunity to collect such samples in this study, but we will pursue this in the future. Finally, we do not know whether some of the clonal groups are still circulating or might have been replaced by new clones. This phenomenon was observed in *S. aureus* where the pandemic clone ST239-MRSA-III that circulated for several decades (Monecke et al., 2018) was replaced with clones with increased fitness (Hsu et al., 2015). Still, we believe that our current study has identified important features of hospital adapted *S. haemolyticus* clones. Future sampling of both commensal and invasive isolates is needed in order to monitor the evolution of *S. haemolyticus*.

## Conclusion

In this study we have gained a deeper understanding of the mechanism of adaption of *S. haemolyticus* in the hospital environment by phylogenetic and pan-genome analysis. We have found a clear segregation of isolates of commensal origin, and specific genetic signatures distinguishing the clinical isolates from the commensal isolates. It is highly likely that the widespread use of antimicrobial agents has promoted the development of MDR clones of *S. haemolyticus* persisting in the hospital environment, and that these isolates have responded through acquisition of mobile genetic elements or beneficial point mutations and rearrangements in surface associated genes. Defining pathogen-associated signatures is an important step in infection control. Continuous surveillance and molecular typing are important in order to monitor the spread and evolution of the *S. haemolyticus* hospital clones in the future.

## Data Availability

The datasets generated for this study can be found in the European Nuclotide Archive (www.ebi.ac.uk/ena) study accession no. ERP000943 for the clinical isolates and ERP114853 for the commensal isolates. The DNA accession number for the individual sequences are found in Supplementary Data S2.

## Author Contributions

MP organized and performed the bioinformatic analysis, took part in the study design, wrote the first version of the manuscript, and revised the manuscript. EH participated in bioinformatical analyzes and revised the manuscript. JC and CK conceptualized and designed the study and revised the final manuscript. All authors approved the final manuscript as submitted and agreed to be accountable for all aspects of the work.

## Conflict of Interest Statement

The authors declare that the research was conducted in the absence of any commercial or financial relationships that could be construed as a potential conflict of interest.

## Footnotes

1 http://www.eucast.org/

2 www.ebi.ac.uk/ena

3 https://itol.embl.de/

4 http://pubmlst.org

5 http://enterobase.warwick.ac.uk

6 http://bacdb.org/BacWGSTdb

7 https://pubmlst.org/shaemolyticus/

## Abbreviations

ARG: antibiotic resistance genes
aSec: accessory secretion system
CARD: comprehensive antibiotic resistance database
CDS: coding sequence
COG: cluster of orthologous groups
CoNS: coagulase negative staphylococci
CP: capsule polysaccharide
ENA: European Nucleotide Archive
IS: insertion sequences
MDR: multi drug resistant
Orf: open reading frame
WGS: whole genome sequence.
